# High-Precision Determination of the Neutron Coherent Scattering Length

**DOI:** 10.6028/jres.110.031

**Published:** 2005-06-01

**Authors:** Apoorva G. Wagh, Sohrab Abbas

**Affiliations:** 86 Dhruva, Solid State Physics Division, Bhabha Atomic Research Centre, Mumbai 400085, India

**Keywords:** coherent scattering length, neutron interferometry, nondispersive phase

## Abstract

The neutron coherent scattering length *b*_c_ has been determined interferometrically to an uncertainty of about 5 × 10^−5^ by measuring the nondispersive phase. We propose improving the uncertainty to about 10^−6^ by optimizing various parameters of the interferometric experiment. Any uncertainty in the *b*_c_ determination arising from possible variations in the constitution of the ambient air can be eliminated by performing the experiment in vacuum. When such uncertainty is attained, it becomes necessary to account for the neutron beam refraction at the sample-ambient interfaces, to infer the correct *b*_c_ from the observed phase. The formula for the phase used hitherto is approximate and would significantly overestimate *b*_c_. The refractive index for neutrons can thus be determined to a phenomenal uncertainty of about 10^−12^.

## 1. Introduction and Discussion

The coherent scattering length for neutrons is an important parameter that describes the neutron-nuclear interaction. A precise knowledge of coherent scattering length is important for understanding the basic nucleon-nucleon interaction, charge independence and charge symmetry of the nuclear forces. Precise determination of *b*_c_ is also needed for different isotopes for material science applications [1,2]. There are many techniques such as Christiansen filter, gravity refractometer, mirror reflection, prism reflection, pendellösung oscillation etc. [1,2] for *b*_c_ determination. Shull et al. [3] attained the least uncertainty of about 0.03 % in *b*_c_ determination of silicon by observing the pendellösung oscillations.

Perfect crystal interferometry affords precise determination of the coherent scattering length [1-2] of samples. With a parallel-faced sample slab of thickness *D* and atomic density *N*, placed normal to one subbeam in the interferometer, neutrons acquire the phase

$$\Phi =-(Nb_{c}-N_{a}b_{a})D\lambda .$$ (1)

Here *λ* denotes the incident neutron wavelength and symbols with the subscript a stand for the corresponding properties of ambient air or vacuum. This variation of *Φ* with *λ* over the spread Δ*λ* in the incident wavelengths reduces the interference contrast. The consequent loss in phase precision limits the attainable *b*_c_ uncertainty to about 10^−3^.

**Fig. 1:**
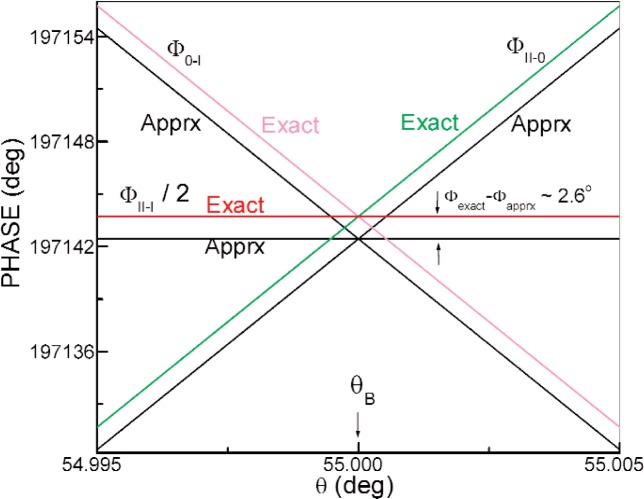
Exact and approximate nondispersive phases in air.

Rauch et al. [4] reduced the uncertainty to about 4.7 × 10^−4^ by following Scherm’s suggestion to insert the sample with its surface parallel to the Bragg planes of the interferometer. Neutrons of each wavelength from the beamsplitter are then incident at the corresponding Bragg angle *θ*_B_ to the sample and the phase

$$\Phi \approx -(Nb_{c}-N_{a}b_{a})D\lambda /\sin\theta_{B}=-2(Nb_{c}-N_{a}b_{a})Dd,$$ (2)

is *nondispersive*. Here *d* symbolizes the Bragg planar spacing. However, here the phase varies sharply with the inclination *θ* of the sample (cf. *Φ*_0–I_ and *Φ*_II−0_ curves in Fig. 1). The nondispersivity condition therefore requires the sample to be aligned with arcsecond precision.

**Fig. 2:**
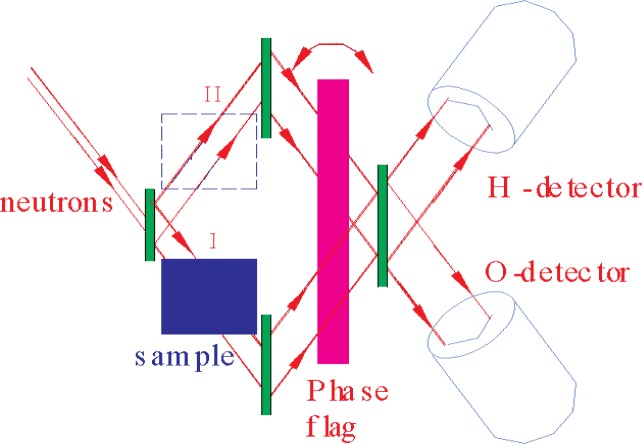
Our proposal.

Ioffe et al. [5] overcame this limitation by measuring the phase shift between interferograms recorded with the sample placed alternately in subbeams I and II (Fig. 2). This eliminates the first order variation of the phase (cf. *Φ*_II–I_ curve in Fig. 1) with the horizontal misalignment Δ*θ* from *θ*_B_. The sample alignment thus requires only arcminute precision to locate the minimum in *Φ*_II–I_, occurring at the intersection of *Φ*_0–I_ and *Φ*_II–0_ curves. The nondispersive phase shift

$$\Phi_{\text{I-II}}\approx -\frac{(Nb_{c}-N_{a}b_{a})Dd}{\cos\Delta \gamma }2(2+{\left(\Delta \theta \right)}^{2}\{1+2\cot^{2}\theta_{B}\}),$$ (3)

then determines the coherent scattering length

$$b_{c}\approx -\frac{\Phi_{\text{I-II}}\;\cos\Delta \gamma }{4NDd\left(1+\frac{\Delta \theta^{2}}{2}(1+2\cot^{2}\theta_{B})\right)}+\frac{N_{a}b_{a}}{N},$$ (4)

Δ*γ* denoting the vertical misalignment of the sample. The experiment [5] achieved a precision Δ*b*_c_/*b*_c_ of 5.1 × 10^−5^, whose source-wise constituents are listed on the left hand side of Table 1. By far the most predominant contribution arises from the relative variation Δ*D*/*D* in the sample thickness.

**Fig. 3:**
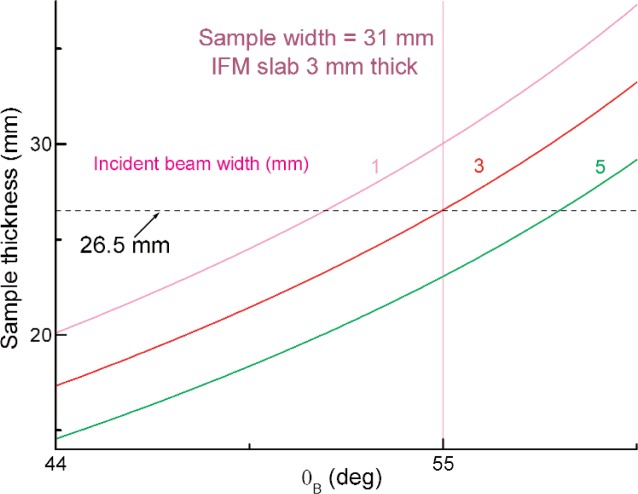
Variation of the allowed sample thickness with Bragg angle.

The uncertainty can hence be lowered by increasing *D* and reducing its variation Δ*D*. An increase in *D* dictates a large Bragg angle (Fig. 3). For practical reasons, we limit *θ*_B_ to 55° (Fig. 2) allowing *D* = 26.5 mm for a 3 mm wide incident neutron beam. The width of the interferometer becomes rather large, about 12.5 cm, at this *θ*_B_. Attaining Δ*D* = 0.1 μm with a precision grinding and polishing machine would yield about an order of magnitude reduction in the Δ*D*/*D* contribution to Δ*b*_c_/*b*_c_. In addition, the phase also increases by the same factor as *D*, reducing the Δ*Φ*/*Φ* contribution. The corresponding neutron path length of 32.4 mm within the sample would still yield a good interference contrast as observed by Rauch et al. [4]. Further, we can maximize *d* to 0.314 nm by choosing the {111} Bragg reflection for the interferometer (hence *λ* = 0.514 nm) to further enhance *Φ* and reduce Δ*Φ*/*Φ*. A thermal enclosure around and vibration isolation of the interferometer reduces the phase drift to a fraction of a degree over a day [2,5]. The effect of this phase drift over a typical measurement duration of a few hours, is minimized by recording the O and H detector intensities (Fig. 2) for the three positions (I, II, and Out) of the sample in succession at each angular setting of the phase flag. A phase error of about 0.3°, thus routinely achieved in interferometric experiments, is included in Table 1. The contribution from the uncertainty in the refractive index of air, dependent on variations in the temperature, pressure and relative humidity, can be larger than that assumed in [5], viz., *N*_a_*b*_a_/*N* = (9.137 ± 0.009) × 10^−3^ fm. This can be eliminated by performing the experiment in vacuum. With a crystalline silicon sample (*Nd* = 1.57 × 10^15^ cm^−2^), our proposed phase *Φ*_I–II_ = − 394284.8° will yield *b*_c_ with uncertainties as shown on the right hand side of Table 1.

When such uncertainty is achieved, it becomes necessary to account for neutron refraction at the ambient sample interfaces. Conservation of the tangential component of the neutron wave vector across the interface yields the exact phase

$$\begin{array}{l} \Phi_{\text{I-II}}=\frac{4\pi D}{\lambda }\left(\sqrt{n^{2}-n_{a}^{2}\;\cos^{2}}\theta_{B}-n_{a}\;\sin\;\theta_{B}\right)\\ \;=4\pi D\left(\sqrt{\frac{-(Nb_{c}-N_{a}b_{a})}{\pi }+\frac{n_{a}^{2}}{4d^{2}}}-\frac{n_{a}}{2d}\right), \end{array}$$ (5)

*n* denoting the refractive index. The exact and approximate [Eq. (3)] phases for Δ*γ* = 0 in our proposal are plotted in Fig. 1. The exact phase is greater by about 2.6° at *θ* = *θ*_B_. The exact phase [Eq. (5)] is rigorously nondispersive only in vacuum, i.e., when *n*_a_ = 1. However, since the refractive index of air differs from unity only by about 1.4 × 10^−8^, the phase is nondispersive to an excellent approximation even in air, to better than 3 × 10^−10^ for an incident wavelength spread Δ*λ* / *λ* of 1 %. Equation (5) yields the coherent scattering length

$$b_{c}=\frac{n_{a}\Phi_{\text{I-II}}}{4NDd}-\frac{\Phi_{\text{I-II}}^{2}}{16\pi ND^{2}}+\frac{N_{a}b_{a}}{N}.$$ (6)

Therefore the correction to the inferred *b*_c_ due to the refraction effects

$$\frac{\Delta b_{c}}{b_{c}}\approx -\frac{Nb_{c}d^{2}}{\pi }=-6.5\times 10^{-6},$$

slightly exceeds the proposed precision in magnitude, underscoring the importance of refraction effects.

The refractive index, *n* = (1 − *Nb*_c_*λ*^2^/π)^½^ of silicon for thermal neutrons equals unity to within about 1 × 10^−6^. Our proposal can thus determine the refractive power, *n* – 1 ≈ 10^−6^, with a relative uncertainty of about 10^−6^, and hence the refractive index to a phenomenal uncertainty of about 10^−12^.

In conclusion, we have proposed an optimized interferometric measurement of the nondispersive phase to determine the neutron coherent scattering length of silicon to an uncertainty of 4 × 10^−6^.

## Figures and Tables

**Table 1:** Comparison between various Δ*b*_c_/*b*_c_ contributions at present [5] and in the proposed experiment

Present	Source	Proposed	
5.0 × 10^−5^	Thickness: Δ*D* → 0.1 μm (Precision grinding)	3.8 × 10^−6^	
9.0 × 10^−6^	Phase: Δ*Φ* = 0.3°, typical	7.6 × 10^−7^	1.2 × 10^−6^
		[111]	[220]
2.2 × 10^−6^	Air: Δ(*N*_a_*b*_a_/*N*) = 9.0 × 10^−6^ fm		2.2 × 10^−6^
	↓		
	Eliminate → Vacuum expt		
1.1 × 10^−7^	Δ*θ* ≈ 0.01°, typical	3.0 × 10^−8^	
1.4 × 10^−7^	Δ*γ* ≈ 0.01°, typical	1.5 × 10^−8^	
3.7 × 10^−9^	Δ{*Nd*}_Si_ = 6 × 10^6^ cm^−2^	3.7 × 10^−9^	
5.1 × 10^−5^	Total	4.4 × 10^−6^	4.5 × 10^−6^
		[111]	[220]
	Vacuum expt	3.9 × 10^−6^	4.0 × 10^−6^
		[111]	[220]
